# A novel approach for discovering stochastic models behind data applied to El Niño–Southern Oscillation

**DOI:** 10.1038/s41598-021-81162-2

**Published:** 2021-01-29

**Authors:** Roman Olson, Soon-Il An, Soong-Ki Kim, Yanan Fan

**Affiliations:** 1 Irreversible Climate Change Research Center, Yonsei University, Seoul, South Korea; 2 Department of Atmospheric Sciences, Yonsei University, Seoul, South Korea; 3 School of Mathematics and Statistics, UNSW Sydney, Sydney, Australia

**Keywords:** Physical oceanography, Statistics, Applied mathematics

## Abstract

Stochastic differential equations (SDEs) are ubiquitous across disciplines, and uncovering SDEs driving observed time series data is a key scientific challenge. Most previous work on this topic has relied on restrictive assumptions, undermining the generality of these approaches. We present a novel technique to uncover driving probabilistic models that is based on kernel density estimation. The approach relies on few assumptions, does not restrict underlying functional forms, and can be used even on non-Markov systems. When applied to El Niño–Southern Oscillation (ENSO), the fitted empirical model simulations can almost perfectly capture key time series properties of ENSO. This confirms that ENSO could be represented as a two-variable stochastic dynamical system. Our experiments provide insights into ENSO dynamics and suggest that state-dependent noise does not play a major role in ENSO skewness. Our method is general and can be used across disciplines for inverse and forward modeling, to shed light on structure of system dynamics and noise, to evaluate system predictability, and to generate synthetic datasets with realistic properties.

## Introduction

Stochastic differential equations (SDEs) are used to model phenomena from a variety of disciplines ranging from finance to hydrology, from rotational diffusion in granular media to climatology^1–13^. A key problem is estimating these equations from available observed time series data, with subsequent modeling and prediction^1–8,14–17^. The core problem can be stated as follows: given a time series ${\mathbf {y}}^{(t)}, t=1, \dots , n$ observed at *n* time points where ${\mathbf {y}}^{(t)} \in {\mathbb {R}}^D$, find the underlying stochastic dynamical model:

1 $$\begin{aligned} \frac{d {\mathbf {y}}}{dt} = {\mathbf {a}}({\mathbf {y}}) + {\mathbf {B}}({\mathbf {y}}) \varvec{\xi _0} = {\mathbf {a}}({\mathbf {y}}) + \varvec{\xi }({\mathbf {y}}), \end{aligned}$$

where **a** is a vector mean function ${\mathbb {R}}^D \rightarrow {\mathbb {R}}^D$ (sometimes called drift coefficient), $\varvec{\xi _0}$ is a vector of zero-mean noise with standard deviation of one (typically assumed to be standard independent Gaussian noise) and ${\mathbf {B}}$ is a matrix-valued diffusion coefficient (a function ${\mathbb {R}}^D \rightarrow {\mathbb {R}}^{D \times D}$). Here, we call $\varvec{\xi }({\mathbf {y}})$ the noise function. It is a random variable whose probability density function (pdf) depends on state ${\mathbf {y}}$. We are going to assume that its components (e.g., $\xi _i$ and $\xi _j$, $i \ne j$) are independent conditional on ${\mathbf {y}}$. We note that the word “noise” does not necessarily represent a diffusion process, it can be any external physical process that is forcing the system and that is not modelled directly via a state variable.

Recent work in the area has broken important new ground, yet it makes assumptions about the pdf of the noise function or the structure of the mean function^1–6,8,15,18^. This limits the generality of these approaches. Here we present a novel and a flexible method that makes no parametric assumptions about the SDE. Notably we do not restrict the noise function $\varvec{\xi }({\mathbf {y}})$ to be Gaussian or independent in time. We approach this problem from a purely statistical perspective. We note that equation (1) can be represented in a probabilistic way by specifying that the state derivative is a *D*-dimensional random variable with a conditional distribution given the state as:

2 $$\begin{aligned} p \left( \frac{d{\mathbf {y}}}{dt} | {\mathbf {y}} \right) , \end{aligned}$$

or for a particular component *i* of the state:

3 $$\begin{aligned} p \left( \frac{dy_i}{dt} | {\mathbf {y}} \right) . \end{aligned}$$

If the joint probability density function (pdf) of $p \left( {\mathbf {y}}, \frac{d y_i}{dt} \right)$ is known, the conditional probability can be obtained as simply:

4 $$\begin{aligned} p \left( \frac{dy_i}{dt} | {\mathbf {y}} \right) = \frac{p \left( {\mathbf {y}}, \frac{d y_i}{dt} \right) }{p({\mathbf {y}})}. \end{aligned}$$

The main problem is estimating the joint probability $p \left( {\mathbf {y}}, \frac{d y_i}{dt} \right) = p \left( y_1, ..., y_D, \frac{dy_i}{dt} \right)$. Here we estimate this joint pdf from available observed samples of $({\mathbf {y}}, \frac{dy_i}{dt})^{(1)}, ..., ({\mathbf {y}}, \frac{dy_i}{dt})^{(n)}$ using kernel density estimation (KDE). The only parameter that the method requires is a bandwidth matrix. We calculate the joint and conditional probabilities on a fine mesh of ${\mathbf {y}}$ and $\frac{d y_i}{dt}$ values.

Strictly speaking, the mean and the noise functions are not employed in the proposed method. However, they can be diagnosed from the conditional pdfs. For example, the estimate of the mean function $a_i({\mathbf {y}})$ can then be derived as a mean of the conditional pdf on this mesh. Conditional on the state value ${\mathbf {y}}$, the $i$th noise component $\xi _i({\mathbf {y}})$ is then a random variable obtained by subtracting for each ${\mathbf {y}}$ the deterministic conditional mean $a_i({\mathbf {y}})$ from the tendency random variable $\frac{dy_i}{dt}$.

The key difference from previous work is that we do not assume any particular form for the mean function $\textit{a}$ or the noise function $\varvec{\xi }$; in fact we do not use explicit mean or noise functions. Generally, the process also does not need to be Markov.

We apply the new method to fitting a dynamical model of El Niño–Southern Oscillation (ENSO). ENSO is a quasi-periodic tropical Pacific phenomenon sustained through ocean-atmospheric coupling, important through its far-reaching effects throughout the globe^2,3,6,19–32^. Its oscillatory feature is characterized by two phases—El Niño with relative warming of the surface equatorial Pacific, and La Niña with a corresponding cooling. The temperature (commonly taken as average sea surface temperature [SST] anomaly from its seasonal mean in one of the designated regions in equatorial Pacific) oscillations are seasonally-locked (i.e., tend to have the highest variance during boreal winter), come about in irregular periods of 2–7 years, and are asymmetrical. Specifically, El Niño events are on average stronger than La Niña events. Several reasons for this asymmetry have been proposed but their relative importance is under debate or may vary for individual ENSO events^4,22–24,26,32^. Another ENSO property, the so-called “transition asymmetry” is the tendency of El Niño events to quickly turn into La Niñas during the following winter, and for La Niñas to persist for more than one year^26,33^.

One commonly used simple ENSO model includes two variables—SST in the eastern equatorial Pacific, and thermocline depth in the equatorial Pacific^4,30^. While the model has substantial skill at reproducing many observed ENSO features, it does not capture ENSO perfectly. This has lead to doubts whether a two-variable system is sufficient to model ENSO in principle. While many other ENSO models exist e.g.,^34^, the search is on to find an ENSO model that includes the smallest number of variables, while still capturing important statistics or properties of ENSO SST anomalies: seasonal standard deviation, spectrum, pdf, transition asymmetry, etc.

Using the new stochastic dynamical fitting method we attempt to answer the following main questions: (1) Can the empirical stochastic-dynamical model estimation method successfully recover a known mean and noise function in a perfect-case setting? (2) Can ENSO be represented as a low-order dynamical model of eastern equatorial SST and equatorial thermocline depth anomalies? (3) What is the relative contribution of nonlinear dynamics, state-dependent noise, and noise self- and cross-correlations to ENSO skewness? and (4) What is the spectral structure of ENSO forcing?

## Results

### Kernel density-based method to recover dynamical systems from time series data

We assume that we have a stationary time series ${\mathbf {y}}^{(1)}, {\mathbf {y}}^{(2)}, \dots , {\mathbf {y}}^{(n)}$. For each time *t* we can approximate the *i*th component tendency as $\frac{dy_i^{(t)}}{dt} \approx \frac{y_i^{(t+1)}-y_i^{(t)}}{dt}$. In the cases analyzed here we assume that $dt=1$, allowing us to drop this term. We apply our method separately to each component *i*. We calculate joint pdfs $p(y_1,... ,y_D, \frac{dy_i}{dt})$ on a fine mesh of ${\mathbf {y}}$ and $\frac{d y_i}{dt}$ values using KDE^35^. We use the R package **ks** for the estimation^36^. We use bandwidth as $s{\mathbf {K}}$ where *s* is a tunable smoothness parameter and ${\mathbf {K}}$ is the plug-in estimator of the bandwidth matrix^35^. We find conditional pdfs $p\left( \frac{dy_i}{dt} | {\mathbf {y}} \right)$ by scaling the joint pdf at each ${\mathbf {y}}$ mesh point so that it integrates to one over all values of $\frac{dy_i}{dt}$ on our mesh. From this conditional pdf, the mean function and the standard deviation of the noise can be easily obtained.

The same method can be extended to cyclostationary processes where sampling interval is an exact fraction of the period. In this case separate joint pdfs can be found for each phase of the process. For example, in the main ENSO application of the paper we work with monthly ENSO observations. ENSO is a cyclostationary process with the frequency of 1 year.

We test the KDE method to estimate the conditional mean and the conditional standard deviation of the noise in a perfect model framework (Supplementary Note 1, Figs. S1–S6). The details are provided in Supplementary Note 1. Overall, these perfect model experiments indicate that for a relatively small number of data points (59 points), and a 2D model, the method can successfully recover some features of mean and noise functions (Figs. S1–S6). The method is especially good at capturing non-planar behavior of the mean tendency (Figs. S1–S2, S5–S6). By “non-planar” we mean any deviation of the conditional mean tendency from a linear function of the form $E(\frac{dy_i}{dt}|y_1, y_2)=k_1y_1 + k_2y_2$ where *E* is the expectation operator. However, for a planar mean function, it may discover non-existent non-planar features (Figs. S3–S4). Since the non-planar behavior of the derivatives is important theoretically in case of ENSO^4^, we believe the KDE method is suited to our particular case.

### Application to ENSO

#### Dynamical model estimates

We apply the KDE method to construct a probabilistic 2D model of ENSO that includes Niño 3 region SST anomaly *T*, as well as thermocline depth anomaly in the equatorial Pacific *h*. All anomalies are with respect to the climatological annual cycle. To construct the model we use monthly data from 1958 to 2016. SST observations are from ERSSTv5^37^, while the 17-degree isotherm data approximating the thermocline depth has two sources. Years 1958–2010 are from SODAv2.2.4 reanalysis^31^ while later data are from GODAS reanalysis^38^. We obtain a separate model for each month *m*. Thus, our combined model is a collection of monthly submodels (see “Methods”).

**Figure 1:**
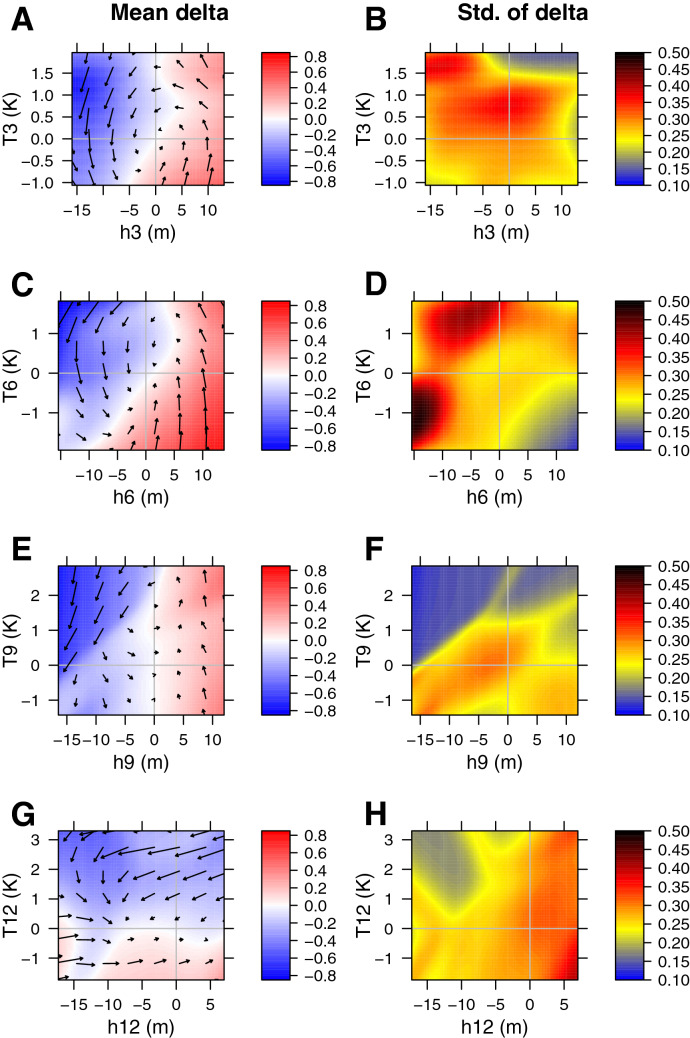
(Left) Mean tendency of SST anomalies in the Niño 3 region for different months (color) [K $\hbox {month}^{-1}$], as a function of Niño 3 SST anomalies *T* [K] and equatorial thermocline depth anomalies *h* [m]. Arrows represent joint tendency of both thermocline depth anomalies [m $\hbox {month}^{-1}$] and SST anomalies [K $\hbox {month}^{-1}$]. (Right) Standard deviation of tendencies of SST anomalies [K $\hbox {month}^{-1}$] as a function of SST and thermocline depth anomalies. Months are (from top to bottom): March, June, September, and December.

We show the mean and the standard deviation of the estimated noise function for SST anomalies in Fig. 1. The ENSO systems exhibits oscillatory behavior, at least for considered months (Fig. 1A,C,E,G). For most of the year, SST response depends primarily on the thermocline, with deeper thermocline forcing surface warming, and shallower thermocline forcing surface cooling. The linear component $\frac{dT}{dt}=c_1 h$ is one of the components of a thermocline feedback^4^, and is known as “angular frequency” in some work. Positive angular frequency was also found in Stein et al.^39^. However, in that work the angular frequency’s magnitude is approximately the same throughout the year, while in our case it appears to vanish in December.

Moreover, there appears to be a non-planar deviation of the mean function for some months. Specifically, in March and June the mean function exhibits rapid changes above a certain SST threshold. This may be a manifestation of possible enhanced response of convection and wind stress to SST anomalies above a certain threshold as previously discussed in Takahashi et al.^40^. We note that any threshold behavior in the real world is not expected to appear as a discontinuity in the mean and noise standard deviation plots because of (i) possible variation of the threshold with time and (ii) smoothing introduced by KDE method. Thresholds of 1.3 and 1.5 K have been mentioned in Takahashi et al.^40^. These are slightly higher than the value of around 1 K found here; note that these estimates are subject to uncertainty and vary with season and for different datasets.

The Bjerknes feedback ($\frac{dT}{dt}=c_2 T$; also known as temperature growth rate) appears to be relatively weak for March, June, and September compared to the thermocline feedback. Our results suggest that Bjerknes feedback is slightly negative in March and June, but is neutral in September. The picture changes drastically in December (Fig. 1G), where negative Bjerknes feedback dominates. This strong negative Bjerknes feedback is a reason for the strong decrease in ENSO standard deviation after December (Fig. 3). The tendency of the Bjerknes feedback to be negative is consistent with the results of Moon and Wettlaufer^41^ and Stein et al.^39^ who also estimate Bjerknes feedback from observations and a high-resolution model hindcast, respectively. They show that this feedback is negative for most seasons, except that it is slightly positive in the fall. These results are also in broad agreement with a physically-based method to calculate Bjerknes stability index^39^.

Since our method may uncover non-planar behavior of the noise standard deviation that is non-existent in the data, we refrain from over-interpretation of the apparent non-planar behavior for some months (Fig. 1B,D,F,H). Specifically, an apparent noise reduction at low thermocline anomalies and high SST anomalies in the fall appears to be caused by a simple lack of observations in that region of phase space (Fig. S7).

ENSO prediction models exhibit a so-called spring predictability barrier in SST^6,7^, broadly defined here as loss of forecast skill when spring is in between the forecast generation and valid times. Our work shows that spring is the period of lowest variability in the modeled SST anomaly (Fig. 3), while overall noise magnitude appears to be similar to other seasons (Fig.  1B,D,F,H). Thus, our results are consistent with the view that the spring barrier is not caused by high absolute noise magnitude, but rather by low signal-to-noise ratio during spring^7^.

**Figure 2:**
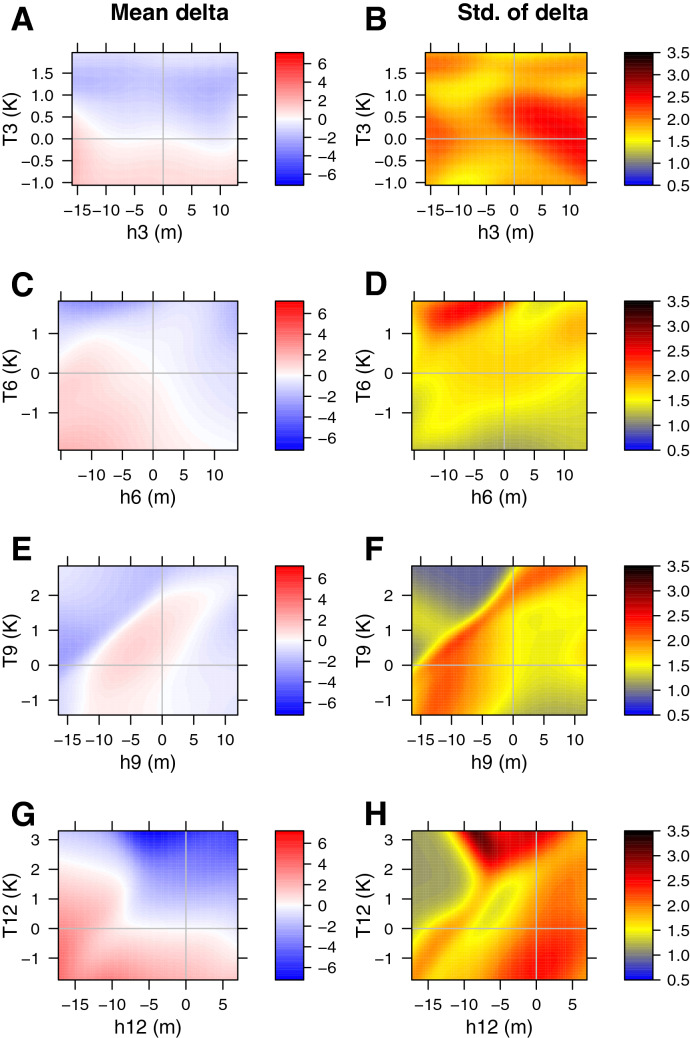
Same as Fig. 1 but for equatorial thermocline depth anomaly tendencies [m $\hbox {month}^{-1}$]. Joint tendency vectors are not shown.

Figure 2 show thermocline tendencies’ conditional means and standard deviations for select months. There is a clear seasonal variability in the mean term, with the strongest mean dynamics in December (Fig.  2A,C,E,G). In particular, in December the therhoclime depth anomaly exhibits a large damping of the form $\frac{dh}{dt}=c_3 T, c_3<0$ in addition to more complex non-planar behavior. This term has been previously called “slow equatorial recharge-discharge process associated with oceanic heat content”^25^. It results from weakening of the trade winds as a response to higher SST anomalies in the Eastern equatorial Pacific. The resulting changes in the wind stress curl induce anomalous Sverdrup transport away from the equator, resulting in anomalous mass and heat divergence in the upper ocean along the equator, thereby shallowing the thermocline. Our results indicate that this process is most important in December. One of the reasons is simply that there is a higher range of observed SST anomalies in December due to increased variability. Another reason may include higher sensitivity of the trade winds to the SST anomalies. Our results suggest a possible non-planar behavior of the mean term, especially in September, when there is a feasible nonlinearity as a function of thermocline depth anomaly (Fig.  2A,C,E,G).

The overall magnitude of the thermocline noise appears to be similar for all seasons (Fig. 2B,D,F,H). Again, we avoid an over-interpretation of the possible non-planarities of the standard deviation of the noise. Specifically, in September there is a noise minimum at low thermocline depth anomalies and high SST anomalies (Fig. 2F). This is, however, an artifact of lack of observations in this region of state space (Fig. S7).

#### Simulation with basic forcing

We note that our dynamical model for *T* and *h* can be formulated as:

**Figure:**
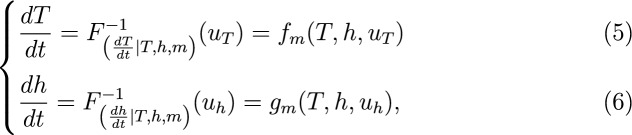

where $F^{-1}_{\left( \cdot |T,h,m \right) }$ is a conditional quantile function of random forcing $u_T$ or $u_h$, mapping this forcing at a given value of *T*, *h* and month *m* to the tendency value. Every tendency value $\frac{dT}{dt}$ is associated, at given *T*, *h*, and *m* with a conditional cumulative distribution function (CDF) value $u_T=F(\frac{dT}{dt} | T, h, m)$ between 0 and 1. This CDF represents the probability that another random sample $\frac{dT}{dt}^{*}$ at the same associated values of *T*, *h*, and month would be below the given $\frac{dT}{dt}$. Thus, $u_T=1$ is associated with SST tendency that is at the extreme upper end of the expected tendencies given the associated *T*, and *h* for the same month. On the other hand, 0 represents an extreme low tendency. $F^{-1}_{\left( \frac{dT}{dt} |T,h,m \right) }$ is a function that maps the CDFs $u_T$ back to the associated tendencies $\frac{dT}{dt}$, given the respective *T*, *h*, and month values. Similar reasoning applies to thermocline tendency, while $f_m$ and $g_m$ are shorthand notations.

The simplest possible forcing of the model is that of uniform independent forcing $u_T$ and $u_h$. Here, however, we use a non-uniform independent forcing, empirically corrected for the overdispersiveness of the stochastic model (e.g., too broad pdf of $\left[ T, h, \frac{dT}{dt} \right] ^T$, see “Methods”).

**Figure 3:**
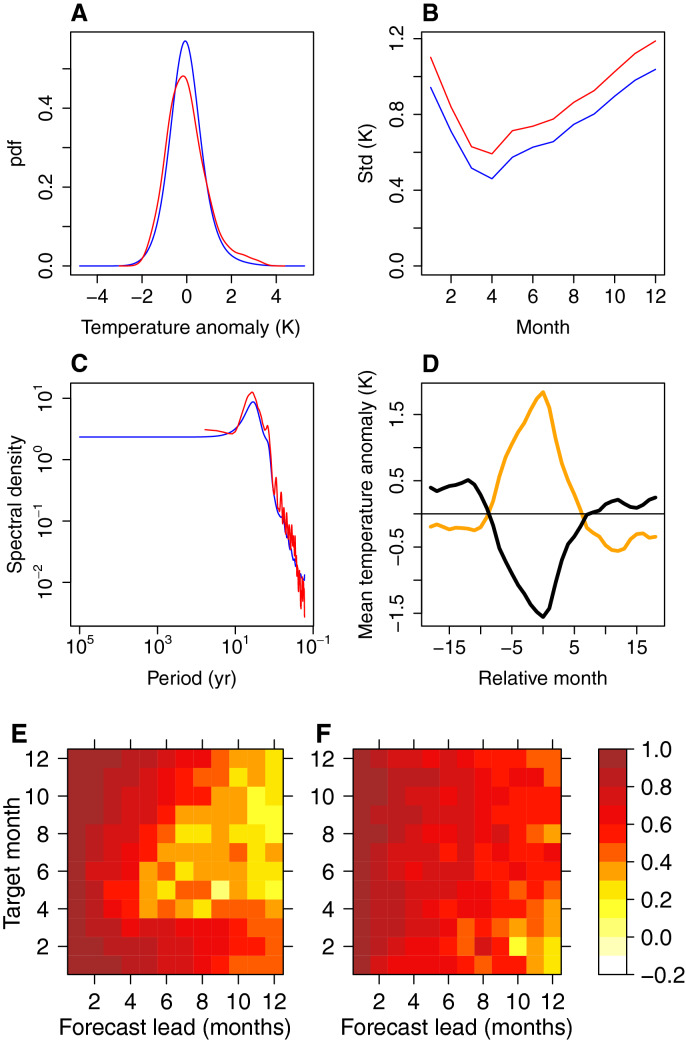
(**A**) Observed (red) and modelled (blue) pdfs of Niño 3 region SST anomalies; (**B**) observed (red) and modelled (blue) standard deviations of the SST anomalies for different months; (**C**) observed (red) and modelled (blue) spectra of the SST anomalies; (**D**) composite plot for modelled SST anomalies for warm (orange) and cold (black) events for months relative to the December of each event; and correlation coefficient between predicted and observed SST (**E**) and thermocline depth (**F**) anomalies for seasons centered on various target months and for different forecast lead times.

Some properties of the associated time series of SST from a stochastic model run forced with such forcing are presented in Fig. 3. The SST anomaly pdf is too narrow (Fig. 3A), and there is a substantial underestimation of the skewness: the observed skewness is 0.84, while modelled skewness is 0.43. A recent work^3^ finds that nonlinear models with quadratic terms could simulate a skewed probability distribution found in the GFDL CM2.1 climate model. However, they use more data by virtue of modelling several principal components of SST, while we only model two variables. Moreover, the skewness in their run of GFDL CM2.1 is much smaller than in the observations and is similar to our empirically modelled value of 0.40.

The simulation with basic forcing can capture the seasonal locking behavior relatively well (Fig. 3B), aside from the lower overall variability. Previous work has identified that seasonally-varying Bjerknes feedback ($\frac{dT}{dt}=c_2(t)T$) is responsible for ENSO seasonal locking behavior^30,39,42^. Moreover, even using a seasonally-resolved 1D model can lead to a good simulation of seasonal cycle^41^. Thus, the ability of our more complex model that includes two variables and a seasonally-varying Bjerknes feedback effect to capture the seasonal locking behavior is quite expected.

There are some minor differences between the modelled and the observed spectra (Fig. 3C). Specifically, the combination mode (C mode) is less pronounced in the modelled spectrum. In addition, the empirical model slightly overestimates the high-frequency variability.

The composite plots of SST anomalies during the cold and warm events are shown in Fig. 3D. Unlike in the observations^26^, there is no marked transition asymmetry between El Niño and La Niña events. The stochastic model also exhibits a spring predictability barrier (Fig. 3E). The thermocline tends to be better predicted at longer lags, but there is also a predictability barrier (Fig. 3F). The barrier is less pronounced compared to SST, and it happens in winter. The winter barrier has been previously identified in literature^43^.

#### Simulation with complex forcing taken from observations

**Figure 4:**
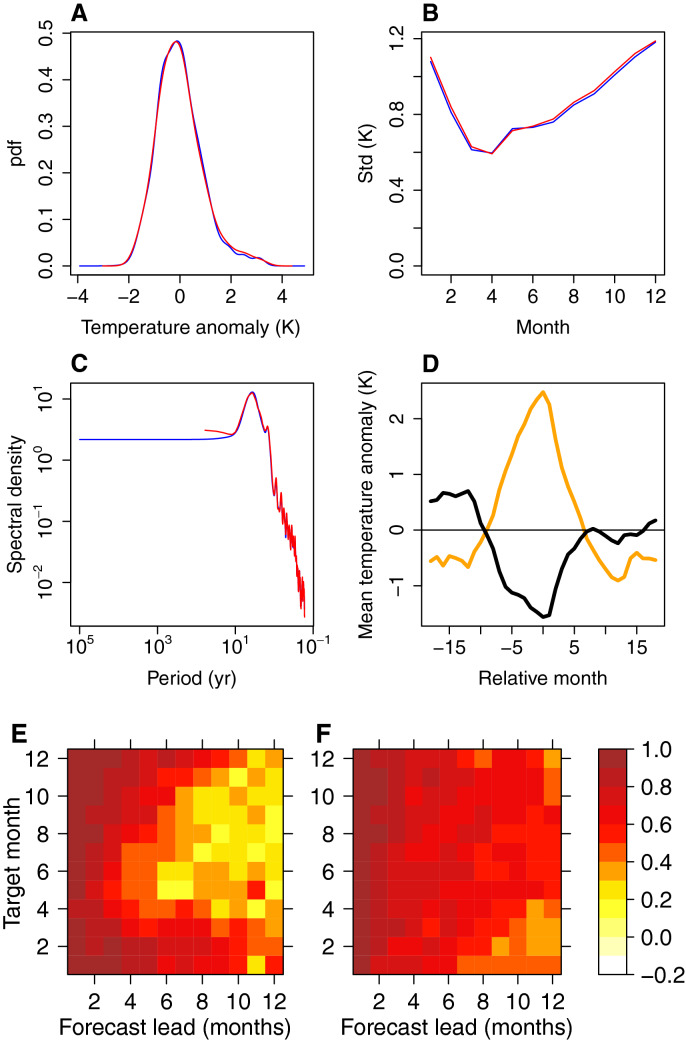
Same as Fig. 3, but for the case of complex time-dependent input forcing that was block-resampled from the observations.

In the more complex forcing case we use empirically derived auto- and cross-correlated $u_T$ and $u_h$ forcing (see “Methods”). The spectra of this forcing are shown in Fig. S8. Our results (Fig.  4) indicate that temporal structure of the forcing increases ENSO amplitude (Fig.  4A,B). This is consistent with previous indications that the component of noise with periods longer than two months is very effective at generating ENSO^44,45^. A range of physical processes can contribute to this forcing component^23,40,46–49^. However, none of the previous work implicates thermocline processes. Our spectral results indicate that there is a previously unidentified oscillation related to the thermocline with a broad period of between about half a year to about two years that may contribute to the ENSO amplitude. The precise role of the different spectral components of the forcing in generating ENSO is a topic of active research^45^. Future work can conduct the sensitivity analysis of the stochastic model to various forms of driving forcing.

Using the more complex forcing dampens short-term variability and improves the representation of the C-mode spectral peak found in the observations (Fig. 4C). The C-mode arises due to the combined effects of annual and interannual frequencies present in the ENSO system^42,50^, and it includes frequencies $1-f$ and $1+f$ where *f* is the natural ENSO frequency. In fact, now the modelled spectrum is in almost perfect match to the observations. Our results suggest that while basic uncorrelated forcing and seasonally-varying dynamics explain most of the spectral features, using more complex forcing provides additional improvements.

The observed pdf is now much better represented, with higher skewness compared to the basic forcing case (Fig. 4A). Specifically, the modelled skewness is now 0.73, which is close to the observed value of 0.84. Choosing even longer block sizes leads to even better skewness results, and this effects holds for empirical models with different smoothness parameters (Fig. S9), and even when model output is nudged to the range of observations (Fig. S10). A block size of 20 years appears to be optimal. However, using larger block sizes decreases effective sample size, since we have a short observational record. Hence, we only analyze a run forced with 10-year resampled forcing in detail. An additional experiment with purely additive independent noise results in skewness of 0.52 (Fig. S11). Here, instead of using uniform CDF noise, we take samples directly from normal distributions with the local conditional means, and standard deviations given by the mean noise standard deviation over the state space for each month. The mean standard deviations are found by an arithmetic average of tendency standard deviations at each grid point of *T* and *h*, without multiplying by their joint pdf.

Previously, ENSO skewness has been explained by a range of physical processes^19,26^, which manifest themselves as (i) the quadratic term of the nonlinear dynamical heating $\frac{dT}{dt}=c_4 T^2$^4^, (ii) threshold behavior of the mean term as a function of SST^40^ or (iii) the temperature-dependent noise^30,44,51^. Our work indicates that the non-planarities in the dynamics (i and ii) explain the majority of ENSO’s skewness, and suggest a relatively small role for the state-dependent noise^52^. On the other hand, the results point to the role of time-dependent forcing in increasing ENSO’s skewness.

Using the more complex forcing also results in transition asymmetry (Fig. 4D). Specifically, an El Niño tends to be followed by a La Niña, yet La Niñas tends to persist until following years, as in the observations^26^. As in the basic forcing case, the stochastic model also exhibits a spring predictability barrier in SST (Fig. 4E). There is no SST predictability improvement from the basic forcing to the more complex forcing case, however the winter thermocline barrier becomes less pronounced (Fig. 4F).

## Discussion

Our method can be compared with previous work^14^. They approximate the conditional pdfs used to calculate the mean and the noise terms with simple histograms. However, this limits their method to large datasets, thus restricting its applicability. In our case we demonstrate that the method can be applied with just 59 data points (for each month).

Our results confirm the view that ENSO can be represented as a low-dimensional system with just two variables, whose dynamics is changing with season, and which is forced by complex external forcing that includes a long-term component. The role of the multiplicative noise in generating ENSO’s skewness appears to be small, if any. Our results suggest that most of ENSO’s observed skewness is a result of feedback processes involving SST and the thermocline, while the rest is contributed to by auto- and cross-correlation in the forcing.

Our work is subject to important caveats. First, we do not provide explicit estimates of the confidence bounds for the mean and the noise functions. Instead, we use the perfect model experiments with known data to qualitatively inform us about the skill of our method. While we provide some sensitivity of our results to the KDE smoothness parameter and to the specifics of the numerical integration (Figs. S9, S10), our estimation of ENSO dynamics needs to be validated using other methods. In addition, the observational record is short. Thus, some of the observed and modelled ENSO time series features may be simply caused by randomness, and are not true features of the system. Finally, this work assumes cyclostationarity of ENSO. In reality, the autocovariance function of SST and thermocline depth anomaly is expected to somewhat vary with time on an interdecadal time-scale due to slow changes in the background state of the tropical Pacific ocean. Moreover, the background state itself can be affected by ENSO through rectification of ENSO variability.

Further work can involve analyzing in greater detail the prediction skill of our model. Ensemble simulations of the stochastic model can provide insights into which features of the observed short observational record are likely to be a real property of the system, and which may arise due to chance. In addition, model simulations with various noise structures can elucidate the effects different noise components have on ENSO properties (e.g., skewness).

In summary, we propose an equation-free method to estimate a stochastic dynamical system from observational data. The method does not impose restrictive assumptions on the data, and allows us to discover the properties of noise which is forcing the underlying dynamical system. We illustrate the skill of the method on three simulated datasets and then apply it to El Niño–Southern Oscillation—a 2-variable system. The method proves that ENSO can be well-represented as two-dimensional system, provides insights into ENSO dynamics, and results in simulations with a near-perfect fit to observed time series properties. This is remarkable given the issues many complex climate models have in simulating these properties^53^ and that these models generally fail at even producing correct ENSO skewness^51^.

The method can be in principle applied across disciplines, with the goal of inverse and forward modelling, and for predictability and prediction research. Furthermore, it could be used for generating large amounts of simulated data with desired properties—a procedure which is relevant for several fields, including health care, fraud detection systems, machine learning, and self-driving cars^54^.

## Materials and methods

### Calculation of SST and thermocline anomalies

Thermocline and SST anomalies are calculated in the following way. First, an area average is carried out for the Niño 3 region (5°S–5°N, 150°W–90°W) and equatorial line for tropical Pacific (120°E–80°W) for SST and thermocline depth (17 °C isotherm), respectively. The isotherm is calculated using vertical linear interpolation of the original ocean temperature data. Both datasets are monthly over a period 1958–2016. Then, the data are linearly detrended, and the seasonal cycle is removed by subtracting monthly means for the entire period. Switching the order of the detrending and seasonal cycle removal has only minor effect on the processed data.

### Details of the empirical stochastic model

We estimate separate models for each month. We use smoothing of $s=1.75$. We choose this value because it results in smooth estimates of the conditional mean and standard deviation fields, while being reasonably close to values used in the perfect model experiments. The KDE estimates are evaluated on a regular $150 \times 150 \times 150$ grid of state and tendency values. The grid encompasses the observed range that has been extended 20% in each direction (e.g., 20% on the bottom and 20% on top) in each dimension.

### Basic forcing

Using uniform forcing $u_T$ and $u_h$ will be incorrect if the stochastic model is overdispersive (e.g., too broad pdf of $\left[ T, h, \frac{dT}{dt} \right] ^T$ at any given month, etc.), which we find is a tendency of our smoothing-based KDE model. Thus, we obtain the forcing empirically as follows: we obtain a sequence of $(u_T, u_h)^{(1)},...,(u_T, u_h)^{(n)}$ in the 59-year long observational and reanalyzed records by calculating the conditional CDF of $\frac{dT}{dt}$ evaluated at the observed sequence $(\frac{dT}{dt}, T, h, m)^{(1)},..., (\frac{dT}{dt}, T, h, m)^{(n)}$, e.g., $u_T^{(i)} = F(\frac{dT}{dt}^{(i)} | T^{(i)}, h^{(i)}, m^{(i)})$. We then take independent samples of $u_T$ and $u_h$ and force the model with these samples (i.e., for each sampled $u_T$ we calculated associated temperature tendency using Eq. 5 and similarly for thermocline). Such re-sampling aims to preserve the overall pdf of $u_T$ and $u_h$, while erasing all self-correlation and cross-correlation at all lags. An independent noise assumption is commonly employed in physically-based simple ENSO models^4,28,39^. We perform a forward integration of the model for 100,000 monthly time steps starting from neutral conditions in January and using Euler’s method.

The relationship between the forcing *u* as used here and the Gaussian noise typically employed in stochastic differential equations (Eq. 1) requires clarification. Previous work often uses standard Gaussian noise $\varvec{\xi _0}$ that is multiplied by a state-dependent matrix-valued diffusion function. Here, similar noise is $\varvec{\xi _0}=[\Phi ^{-1}(u_T), \Phi ^{-1}(u_T)$] where $\Phi ^{-1}$ is a univariate CDF of a standard normal distribution. If the KDE-derived conditional pdf is equal to the true underlying conditional pdf, and in the limit of infinite observations, this noise would follow a standard Gaussian distribution. Note that in our formulation when this noise is zero, the resulting tendency is a conditional median tendency, rather than the conditional mean. We choose to work with the forcing ${\mathbf {u}}=[u_T, u_h]$ rather than with the more similar noise $\varvec{\xi _0}$ since there is a simple transformation between elements of ${\mathbf {u}}$ and the tendencies—the conditional quantile functions $F^{-1}(\cdot )$.

### More complex forcing

In the more complex forcing formulation we account for the fact that the forcing $u_T, u_h$ may be auto- and cross-correlated. We block-resample the previously-obtained empirical 59-year time-series of $(u_T, u_h)^{(1)},...,(u_T, u_h)^{(n)}$, with 10-year block sizes. Such re-sampling aims to preserve forcing autocorrelations, as well as cross-correlations (since blocks of both *T* and *h* are reshuffled together) that are found in the observations. As previously, we run the model for 100,000 years.

### ENSO event definitions

ENSO warm and cold events used to calculate composites shown in Figs.  3 and 4 are defined following prior work^55^, with some differences. Specifically, here a warm event is defined for December when a 5-month running mean of Niño 3 region SST centered on December is above the 90th percentile of the original time-series for three previous months including December (e.g., October, November, and December). The cold events are defined similarly except the running mean SST must be below the 10th percentile.

## Supplementary information

Supplementary information.
