# Supplementary material for: A novel approach for discovering stochastic models behind data applied to El Niño–Southern Oscillation
Source: Sci Rep. 2021 Jan 29;11:2648. doi: 10.1038/s41598-021-81162-2 (PMC7846861; doi:10.1038/s41598-021-81162-2)
Supplement: Supplementary file 1 — Supplementary information. [file 41598_2021_81162_MOESM1_ESM.pdf]

Supplementary Information for: A Novel Approach for Discovering Stochastic Models Behind Data Applied to El Niño - Southern Oscillation

R. Olson, S.-I. An, S.-K. Kim and Y. Fan

December 2, 2020

This supplementary information contains: Supplementary Note 1 with the perfect model tests of the method to estimate stochastic dynamical models, and supplementary figures.

1 Supplementary Note 1

This note describes perfect model tests of the method to recover stochastic dynamical models. We test the KDE method to estimate the conditional mean and the conditional standard deviation of the noise in a perfect model framework. In the first case we assume $p\left(y_1, y_2, \frac{dy_i}{dt}\right)$ is one of a weighted mixture of two 3D multivariate normal distributions. The first distribution, with a weight of 0.3, is a standard independent multivariate normal distribution $N(\mu_1, \Sigma_1)$, where $\mu_1 = (0, 0, 0)$ and $\Sigma_1 = I_3$. The

second component, with a weight of 0.7, is a correlated multivariate normal distribution $N(\mu_2, \Sigma_2)$, where $\mu_2 = (1, 1, 1)$ and

$$\Sigma_2 = \begin{bmatrix} 1 & 0.5 & -0.3 \\ 0.5 & 1.3 & 0.6 \\ -0.3 & 0.6 & 0.7 \end{bmatrix}. \tag{1}$$

We estimate the joint pdf using 59 samples from the distribution (the same amount as used for the real-case ENSO example used later), with $s = 1$. The actual properties are shown in Figure S1, while the estimated properties are in Figure S2. The KDE method captures the overall shape of the 3D marginal pdfs relatively well (Figures S1A-C and S2A-C). Furthermore, the method also recovers the structure of the mean function relatively well (Figures S1D and S2D). Some aspects of the standard deviation of the noise function are also captured, such as the ridge in the upper left part of the phase space (Figures S1E and S2E).

In the second case we consider a standard 3D multivariate normal distribution for data vector $\left[y_1, y_2, \frac{dy_i}{dt}\right]^T$. We use 59 samples as before, and $s = 1.5$. The corresponding results are shown in Figures S3 and S4. We observe that the overall estimated joint pdf shape agrees reasonably well with actual joint pdfs for pairs v_1, v_2 and v_1, v_3, although the estimated pdfs are too diffuse (Figures S3A-B and S4A-B). In addition, the pdfs appear to exhibit a "correlated" structure. This effect arises from the limited number of data samples. More marked departures from the actual joint pdf are found for the pair v_2, v_3 (Figures S3C and S4C). On the unit subspace centered at 0 the deviations of the

conditional mean $a(\mathbf{y})$ from true mean tend to be less than 0.6 (Figures S3D and S4D). The standard deviation of $\xi(\mathbf{y})$ is reasonably close to the actual value of 1 everywhere (Figures S3E and S4E), except the top right corner of the phase space. Large errors in regions away from the origin happen because of lack of data samples there. This is not of concern since that is where the actual pdf of $\mathbf{y}$ is close to 0. Thus, it is unlikely that the system will enter those regions of the phase space.

Figure S5 and S6 present actual and estimated results for a conditional normal distribution which has a nonlinear dependency. The distribution is specified as follows: y_1 and $y_2 \sim N(0, 1)$, $\frac{dy_i}{dt} \sim N(y_2^2, 1)$. This distribution is chosen because of the nonlinearity in the mean tendency as a function of one of the variables. Here, s=1.25 and we use 59 simulated data points, as before. The KDE method has an excellent skill at recovering the mean function (Figures S5D and S6D). The standard deviation is considerably (up to a factor of two) overestimated at large and low y_2 values. Thus, the method captures the nonlinearity in the tendency as a function of y_2 well. Yet, with only a few datapoints available in the whisker regions, the method judges that the high values of the tendency in these regions are partially a result of a high standard deviation (Figures S5E and S6E).

2 Supplementary Figures

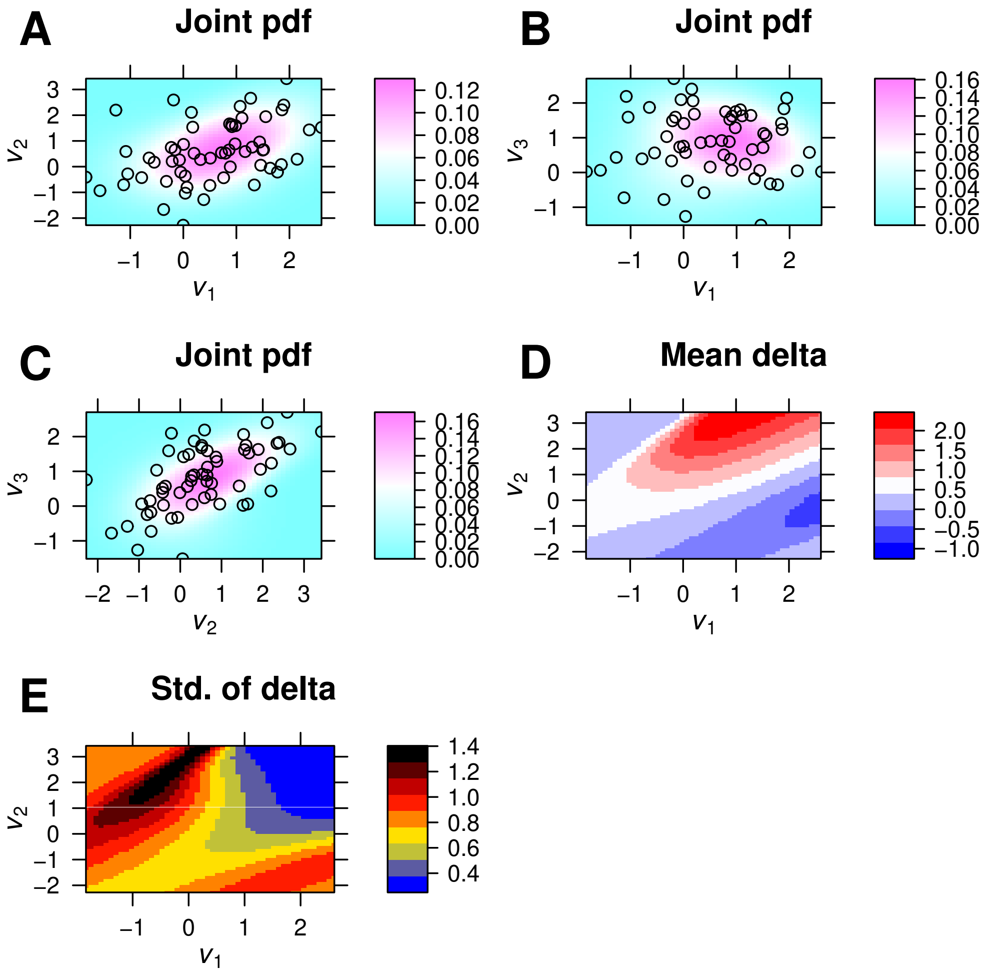

Figure S1: Features of a mixture of two 3D standard normal distribution of a vector $V = [v_1, v_2, v_3]^T = \left[y_1, y_2, \frac{dy_i}{dt}\right]^T$. The third component represents tendency Δ of a variable over time. See text for parameters of the distributions. (**A**), (**B**), (**C**) 2D marginal joint pdfs of pairs of vector components (color), and 59 random samples (circles); (**D**) mean of the conditional distribution $p(v_3|v_1, v_2)$; (**E**) standard deviation of this conditional distribution.

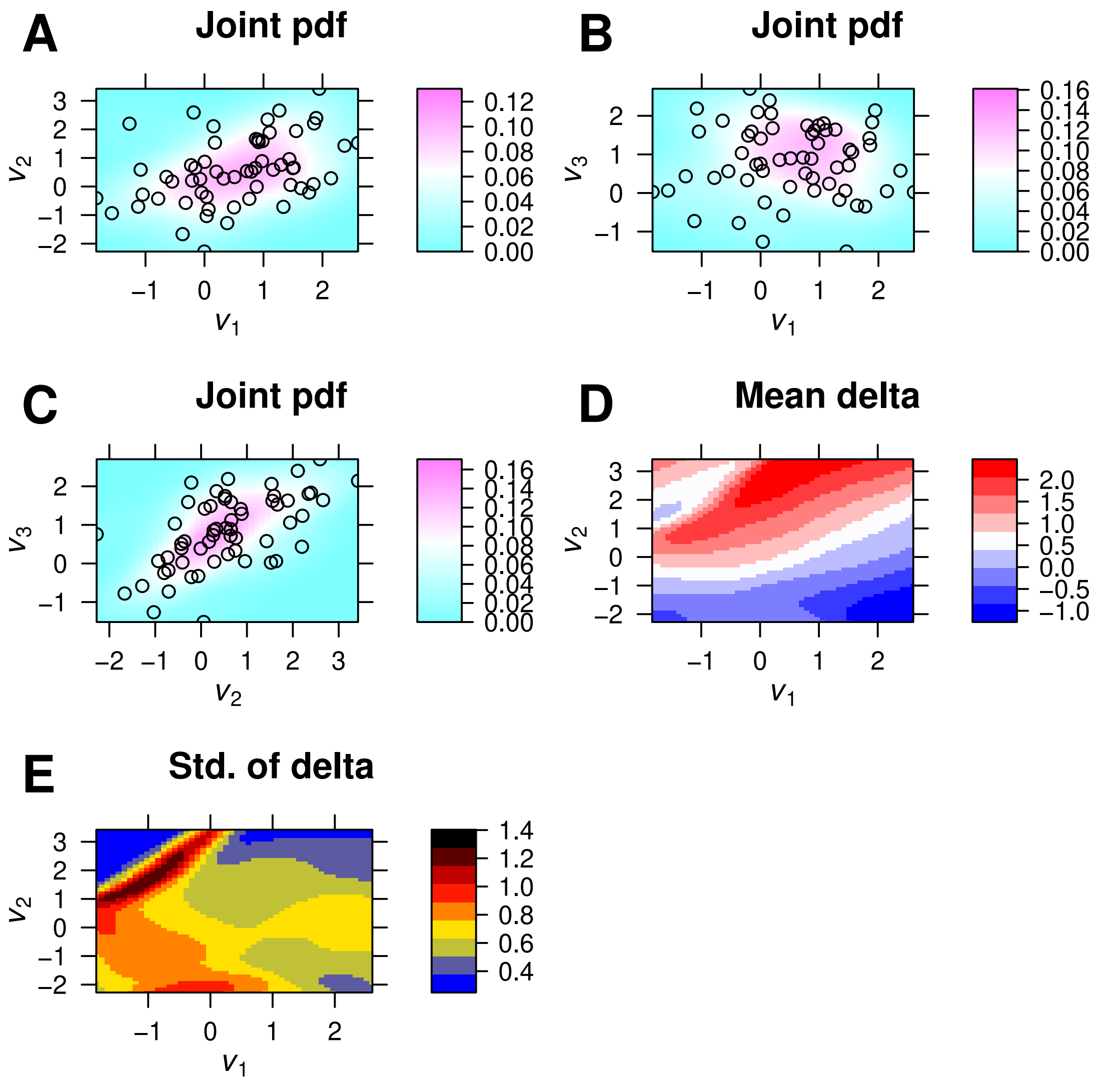

Figure S2: Same as Figure S1, but all pdfs are found using kernel density estimation from the 59 data samples.

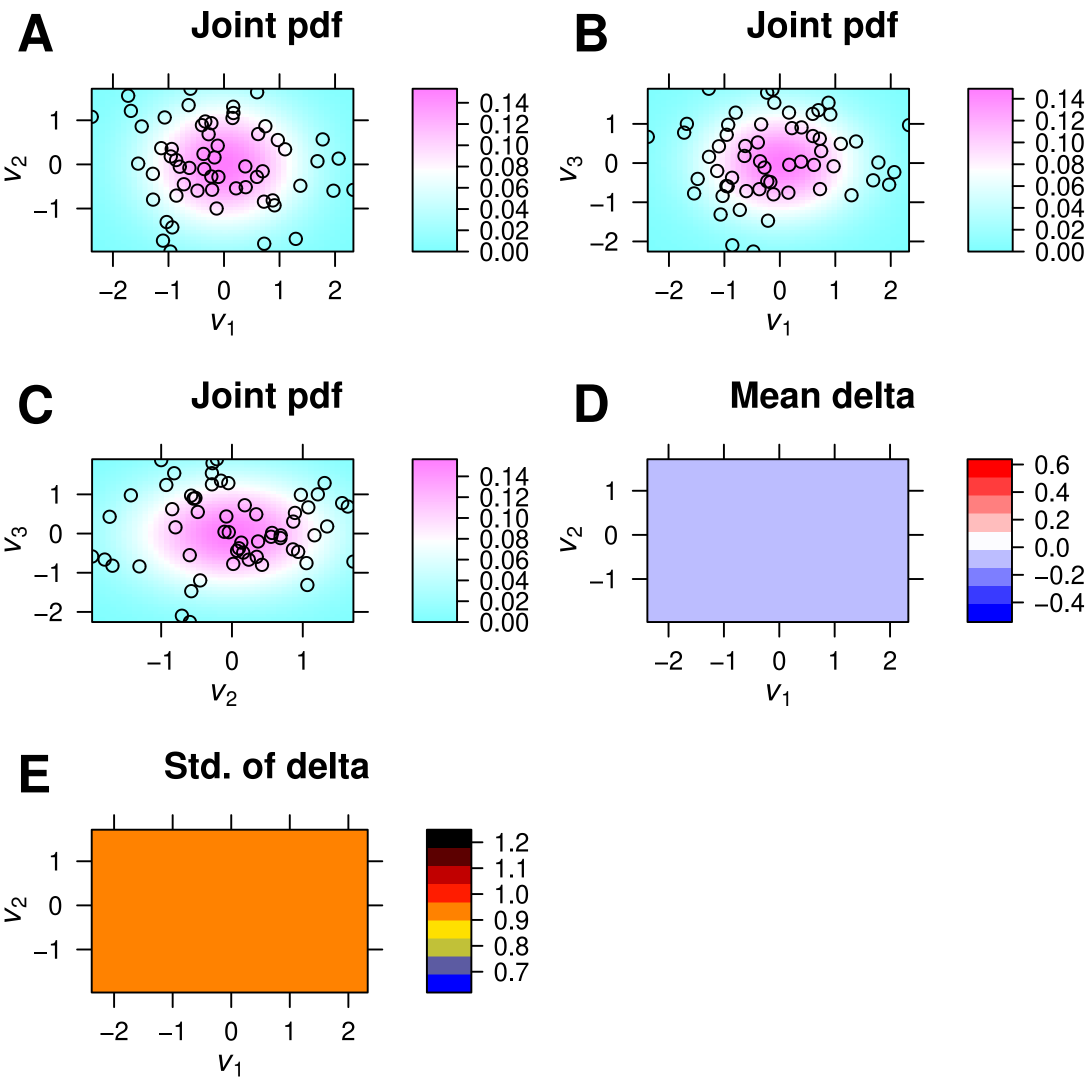

Figure S3: As Figure S1, but for a 3D multivariate standard normal distribution.

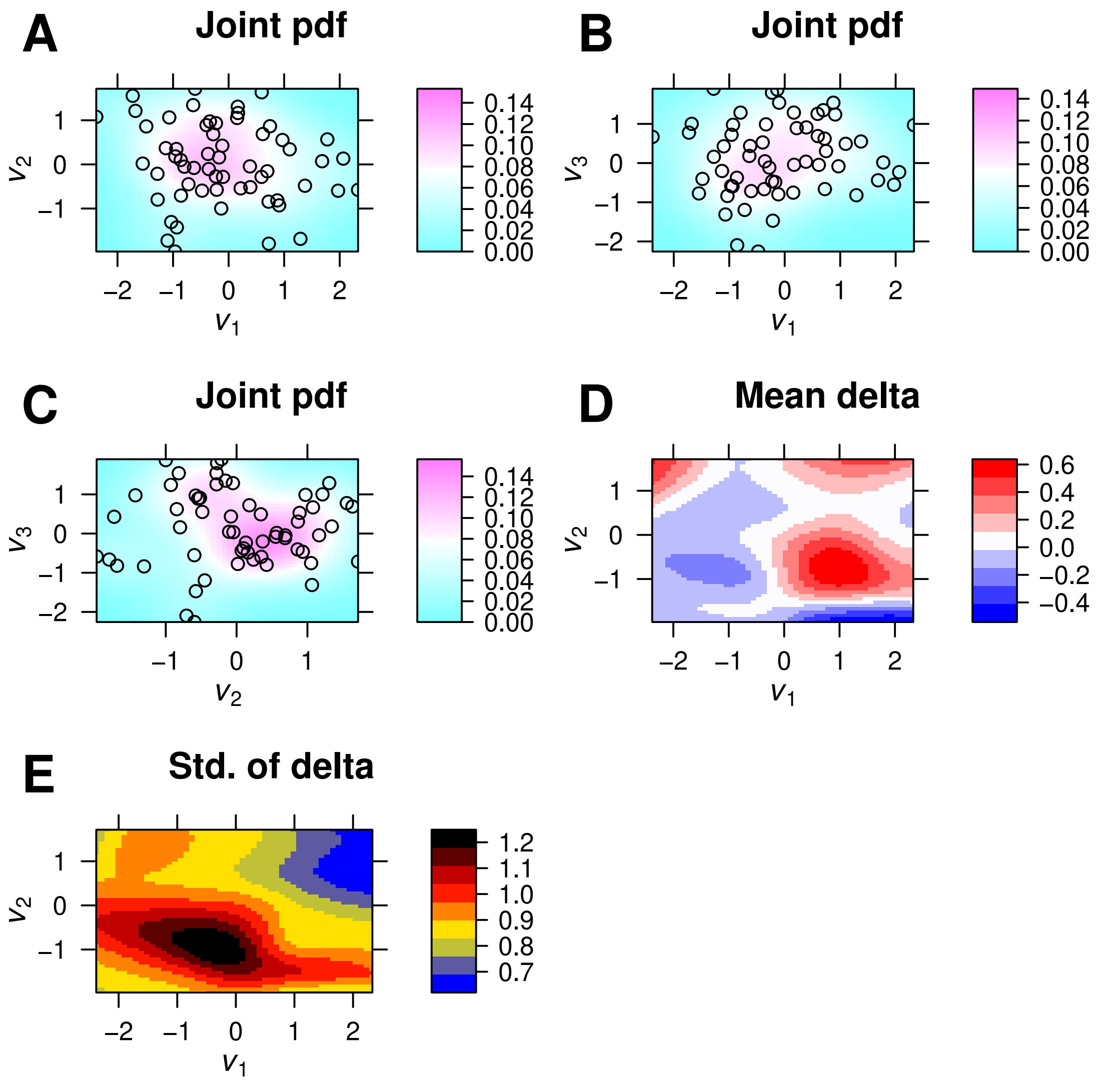

Figure S4: Same as Figure S3, but all pdfs are found using kernel density estimation from the 59 data samples.

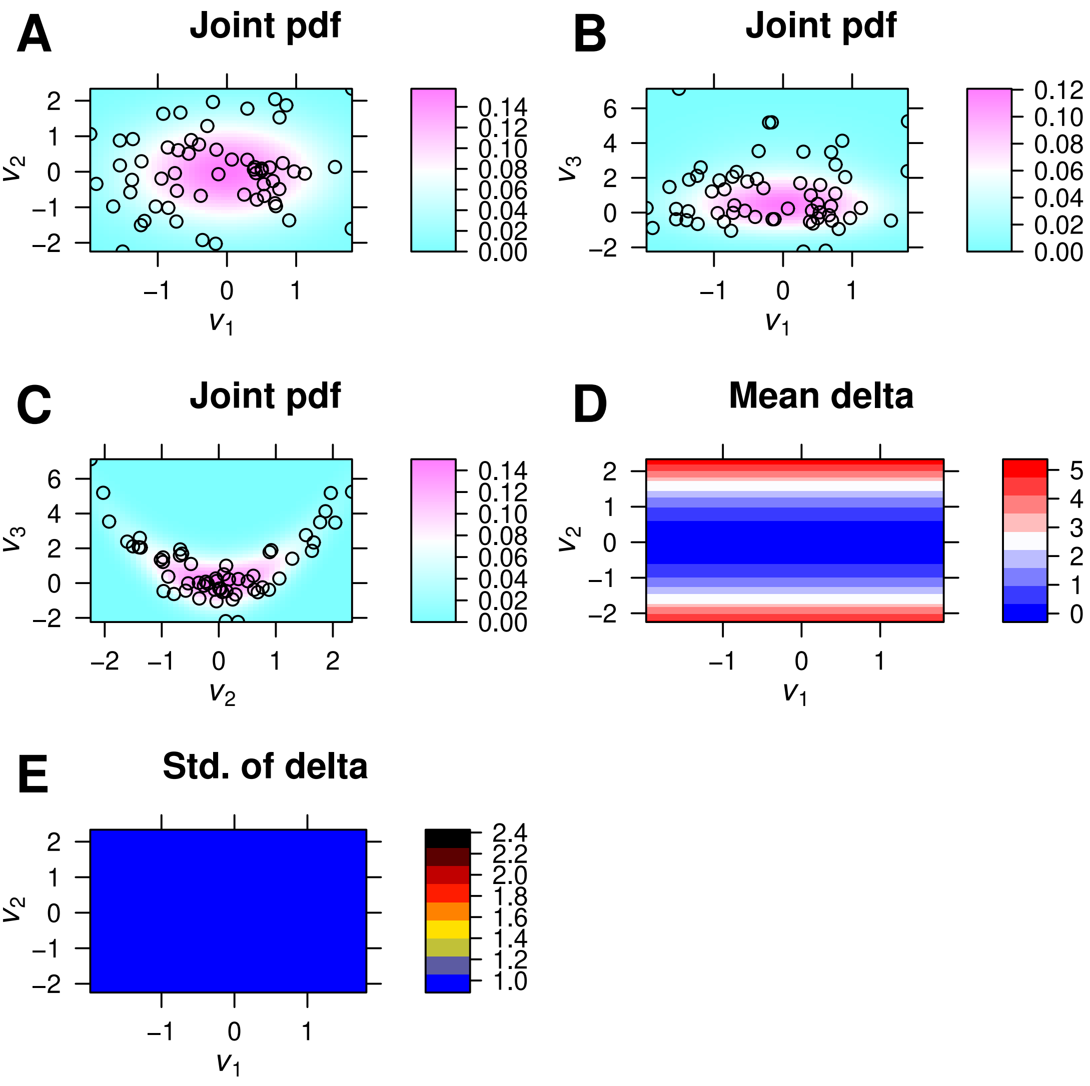

Figure S5: As Figure S3, but for another 3D distribution where v_1 and v_2 are standard normal distributions, and $v_3 = \frac{dy_i}{dt}$ follows a conditional normal distribution. See text for the parameters of the distributions.

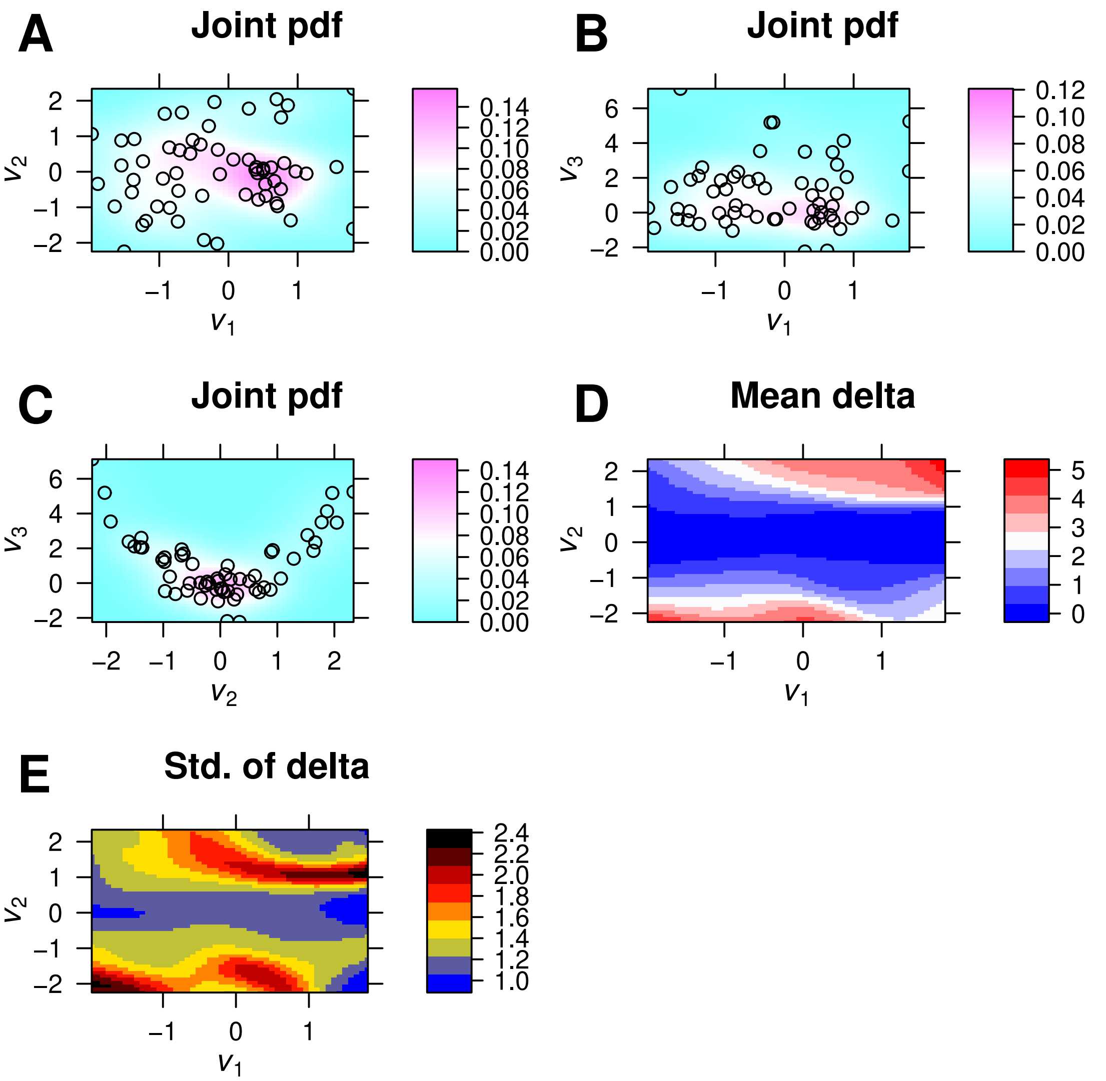

Figure S6: Same as Figure S5, but all pdfs are found using kernel density estimation from the 59 data samples.

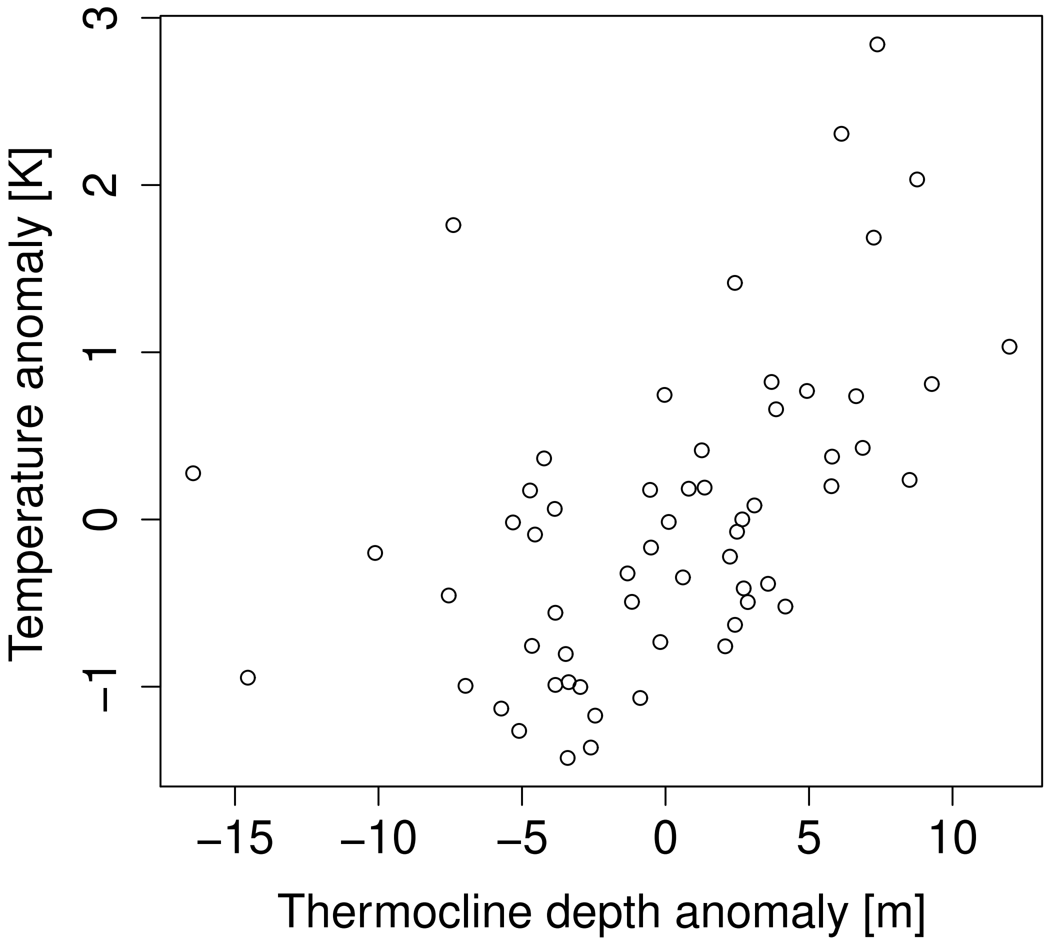

Figure S7: Scatterplot of observed Niño 3 SST anomalies vs. equatorial thermocline depth anomalies for years 1958-2016.

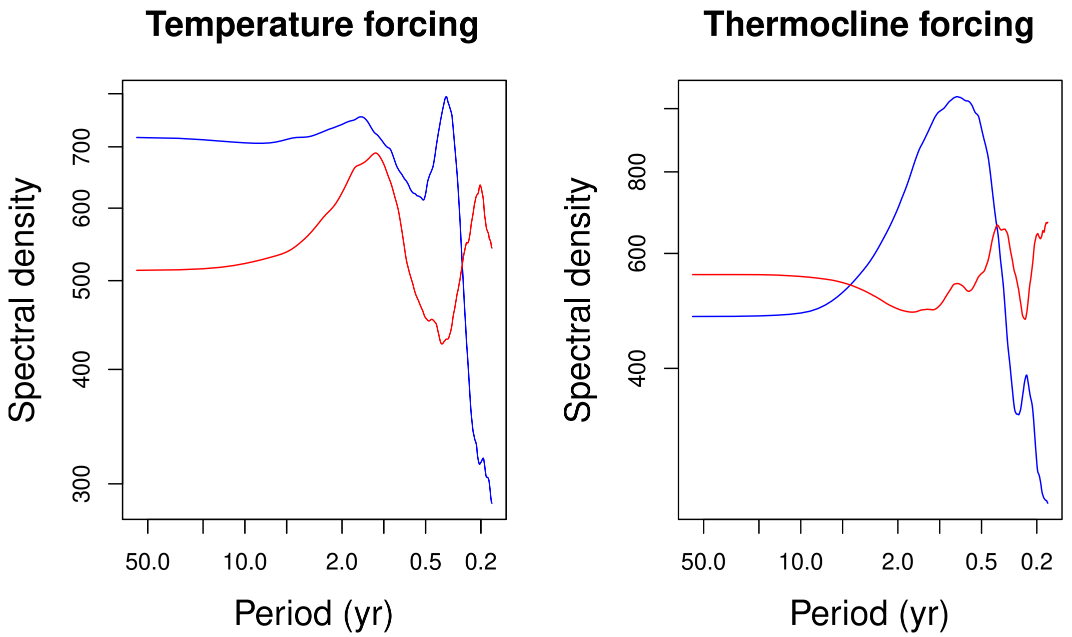

Figure S8: (Left) spectrum of temperature anomaly CDF forcing u_T (blue) found in the observations, compared to the spectrum of a random 59-year realization of uniform forcing (red); (right) the same for thermocline depth anomaly u_h

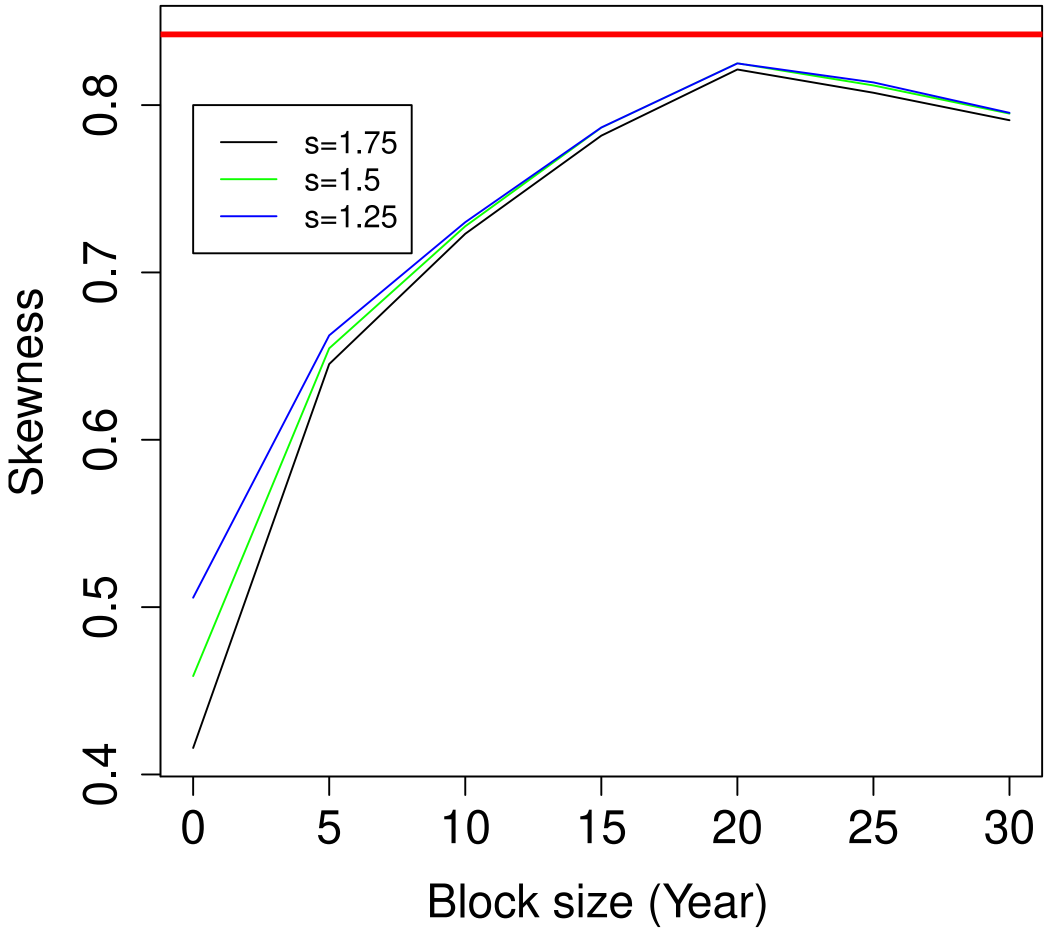

Figure S9: Skewness of observed Niño3 temperature anomalies (red), and that of modelled anomalies where the block size of forcing noise has been varied. Three curves represent stochastic models obtained using different smoothing parameter s.

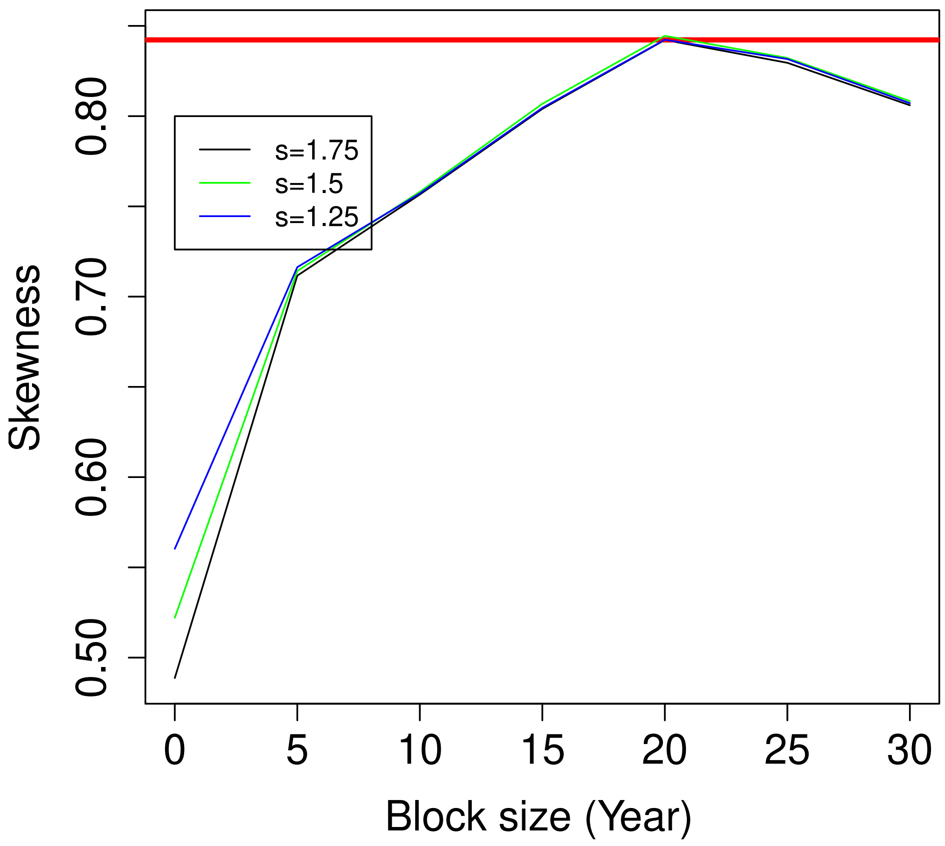

Figure S10: Same as Figure S9 but when model output is linearly pulled towards the observed range for each month when the output is outside this range. Specifically, in these cases if the original model prediction $z_i^{(t)}$ is exceeding observed range for a particular month then the final nudged variable is $z_i^{*(t)} = 0.5 \times max(y_i^{(m_{j_1})}, ..., y_i^{(m_{j_p})}) + 0.5z_i^{(t)}$, where $(y_i^{(m_{j_1})}, ..., y_i^{(m_{j_p})})$ are observed values for that month. Likewise, if the model prediction is below the range, is it transformed using $z_i^{*(t)} = 0.5 \times min(y_i^{(m_{j_1})}, ..., y_i^{(m_{j_p})} + 0.5z_i^{(t)}$. Here z_i is either temperature anomaly or thermocline depth anomaly.

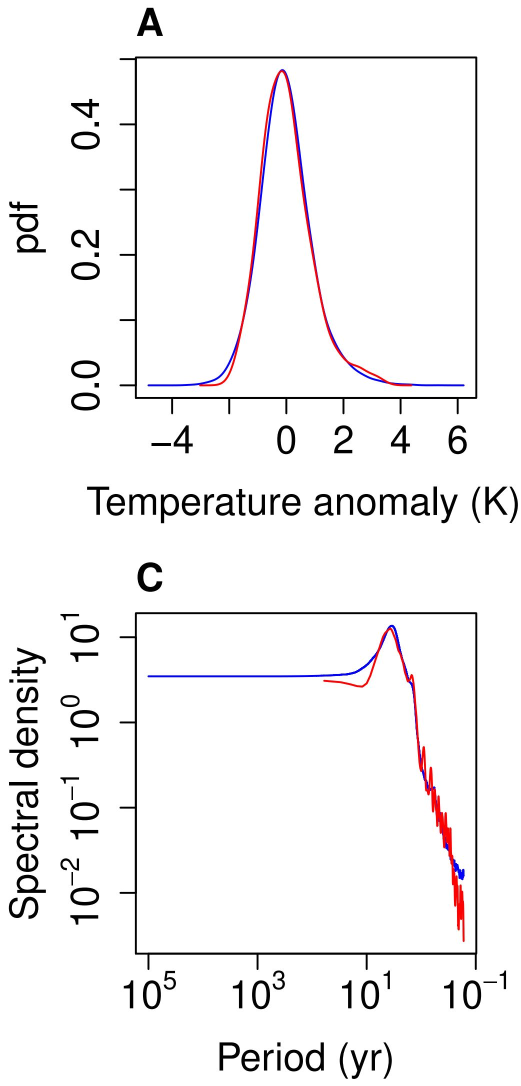

Figure S11: Same as Figure 3 of the main text but excluding the predictability and composites plots, and for additive (e.g., state-independent) noise forcing.
